# Lessons learned from the polio eradication initiative in the Democratic Republic of Congo and Ethiopia: analysis of implementation barriers and strategies

**DOI:** 10.1186/s12889-020-09879-9

**Published:** 2020-12-18

**Authors:** Wakgari Deressa, Patrick Kayembe, Abigail H. Neel, Eric Mafuta, Assefa Seme, Olakunle Alonge

**Affiliations:** 1 School of Public Health, Addis Ababa University, Addis Ababa, Ethiopia; 2 Kinshasa School of Public Health, Kinshasa, Democratic Republic of Congo; 3 International Health Department, Johns Hopkins Bloomberg School of Public Health, 615 N. Wolfe St., Baltimore, MD 21205 USA

**Keywords:** Democratic Republic of Congo, Ethiopia, Implementation science, Knowledge translation, Global Polio Eradication Initiative

## Abstract

**Background:**

Since its inception in 1988, the Global Polio Eradication Initiative (GPEI) has partnered with 200 countries to vaccinate over 2.5 billion children against poliomyelitis. The polio eradication approach has adapted to emerging challenges and diverse contexts. Knowledge assets gained from these experiences can inform implementation of future health programs, but only if efforts are made to systematically map barriers, identify strategies to overcome them, identify unintended consequences, and compare experiences across country contexts.

**Methods:**

A sequential explanatory mixed methods design, including an online survey followed by key informant interviews (KIIs), was utilized to map tacit knowledge derived from the polio eradication experience from 1988 to 2019. The survey and KIIs were conducted between September 2018 and March 2019. A cross-case comparison was conducted of two study countries, the Democratic Republic of Congo (DRC) and Ethiopia, which fit similar epidemiological profiles for polio. The variables of interest (implementation barriers, strategies, unintended consequences) were compared for consistencies and inconsistencies within and across the two country cases.

**Results:**

Surveys were conducted with 499 and 101 respondents, followed by 23 and 30 KIIs in the DRC and Ethiopia, respectively. Common implementation barriers included accessibility issues caused by political insecurity, population movement, and geography; gaps in human resources, supply chain, finance and governance; and community hesitancy. Strategies for addressing these barriers included adapting service delivery approaches, investing in health systems capacity, establishing mechanisms for planning and accountability, and social mobilization. These investments improved system infrastructure and service delivery; however, resources were often focused on the polio program rather than strengthening routine services, causing community mistrust and limiting sustainability.

**Conclusions:**

The polio program investments in the DRC and Ethiopia facilitated program implementation despite environmental, system, and community-level barriers. There were, however, missed opportunities for integration. Remaining pockets of low immunization coverage and gaps in surveillance must be addressed in order to prevent importation of wild poliovirus and minimize circulating vaccine-derived poliovirus. Studying these implementation processes is critical for informing future health programs, including identifying implementation tools, strategies, and principles which can be adopted from polio eradication to ensure health service delivery among hard-to-reach populations. Future disease control or eradication programs should also consider strategies which reduce parallel structures and define a clear transition strategy to limit long-term external dependency.

## Background

Since its inception in 1988, the Global Polio Eradication Initiative (GPEI) and 200 countries have partnered to vaccinate over 2.5 billion children globally against poliomyelitis [1]. The eradication strategy deployed by the GPEI has led to remarkable progress in reducing the burden of poliovirus worldwide. Still, there is increasing global concern over circulating vaccine-derived poliovirus (cVDPV) across the African and Southeast Asian regions and two countries (Afghanistan, Pakistan) remain polio endemic [2]. With the GPEI midway into the 2019–2023 endgame strategic period [3], there is a need to understand the continued threats that countries face in preventing the reemergence of polio, as well as threats to effectively integrating polio assets and institutionalizing lessons learned from the eradication experience. This is especially salient for countries facing key vulnerabilities – e.g. gaps in health system infrastructure, challenges in reaching hard-to-reach populations, and issues related to conflict, political insecurity, and community mistrust – which can exacerbate the risk of outbreak and reinfection.

This paper therefore focuses on implementation of polio eradication activities in two countries in sub-Saharan Africa, the Democratic Republic of Congo (DRC) and Ethiopia, which are classified by the GPEI as “outbreak” countries. Outbreak countries are defined as countries which have halted indigenous wild poliovirus (WPV) but are experiencing reinfection via importation of the wild virus or as is the case here, as a result of cVDPV [4].^1^ While the DRC and Ethiopia’s implementation experiences and contextual challenges are decidedly unique, by systematically linking implementation barriers to change strategies in both contexts, we hope to illuminate key lessons learned which can be generalized and inform ongoing polio eradication strategy as well as future life-saving health programs.

The DRC and Ethiopia joined the GPEI in 1988 and 1996, respectively, and implemented polio eradication using the standard four-pronged polio eradication strategy recommended by the GPEI (routine immunization, supplementary immunization activities, mop-up campaigns in high-risk districts, and surveillance). The polio program has a similar architecture in both countries, even as operational realities vary. In both countries the program is integrated into the expanded program on immunization (EPI) at the operational level and service delivery follows the three-tiered structure of the health system, from peripheral to central levels including primary, secondary, and tertiary or specialized care. Coordination of the program including resource mobilization, logistics management, social mobilization and vaccination are centrally managed via the strategic interagency coordination committees and their technical subcommittees. In both settings, delivery of routine immunization is provided via the EPI and Ministry of Health staff at each level of the system, however, supplementary immunization activities and mop-ups are managed by specialized committees at the national, provincial, district and local levels and require additional staff including vaccinators, supervisors, and data managers, with funding and technical assistance provided by global partners (e.g. the World Health Organization (WHO), United Nations International Children’s Emergency Fund (UNICEF)). In Ethiopia, the CORE Group Polio Project, an extensive network of non-governmental organizations, has also played a critical role in implementing eradication activities among hard-to-reach populations, deploying more than 4000 Community Volunteer Surveillance Focal Persons (CVSFPs) at the village level to conduct house-to-house case detection and reporting of acute flaccid paralysis (AFP) [5]. In the DRC, NGOs are present but have not been engaged as systematically.

Surveillance is managed in the DRC by the EPI with support from the WHO, though the National Institute of Biomedical Research (INRB), a specialized program of the Ministry of Health, is responsible for assessing adequacy of and evaluating stool samples. Since 2009 surveillance functions in Ethiopia have also been managed separately from ministry functions by the Centre of Public Health Emergency Management (PHEM). Based at the Ethiopian Public Health Institute (EPHI), PHEM is an autonomous agency accountable to the Ministry of Health (MOH) which hosts the National Polio Laboratory of Ethiopia. Notably, oversight of laboratory activities (both the DRC and Ethiopia have accredited laboratories within the Global Polio Laboratory Network) is distinct from community-based surveillance. As noted above, a key strategy in Ethiopia has been to conduct active surveillance in remote areas to detect AFP cases via the CORE group network.

Given their varied epidemiologic status (Ethiopia has only recently become affected by cVDPV), the DRC and Ethiopia’s post-polio transition plans are in somewhat different phases. In the DRC, a post-polio transition plan was validated by the government, however it has undergone repeated revisions as a result of the ongoing outbreaks of cVDPV; today, DRC is primarily relying on global-level guidance on transition management, though there remains a lack of consensus on where polio assets should be transitioned to (i.e. to other donor-led initiatives, to the public sector). Ethiopia is currently implementing a five-year transition plan (2018–2022) intended to mainstream polio functions into the national health system over time in order to address the ramp down of GPEI financing. In the current phase (2018–2020) the focus is on strengthening health system capacity prior to institutionalization of polio activities into the government health system, though efforts have also re-shifted toward managing new outbreaks of cVDPV [6].^2^ Transition planning has seen limited and often late-game attention from the GPEI; as a result, there are ongoing questions about the feasibility of institutionalizing public goods from polio eradication while also addressing potentially long-lasting effects of cVDPV.

Considering the implications for maintaining polio-free status and advancing population health, this paper aims to describe and compare barriers to implementation, implementation strategies, and intended and unintended impacts of the GPEI within and across the DRC and Ethiopia. This analysis is part of a multi-phase implementation science project, the *Synthesis and Translation of Research and Innovations in Polio Eradication (STRIPE)* project, which aims to map, package and disseminate key lessons learned from the polio eradication initiative to facilitate effective implementation of public health programs globally [7].

### The Democratic Republic of Congo

The DRC has an estimated population of 84.1 million as of 2019, with urban clusters spread throughout the country, particularly in the northeast along the border with Uganda, Rwanda and Burundi [8]. The DRC has experienced significant periods of violent conflict over the course of the last two decades, including protracted conflicts in 1996 and 1998. The DRC continues to experience violence committed by armed groups including the Democratic Forces for the Liberation of Rwanda, the Allied Democratic Forces, and assorted Mai Mai militias**.** As of August 2018, an estimated 4.5 million Congolese were internally displaced, the vast majority fleeing violence between rebel groups and Congolese armed forces [9]. Half a million refugees have also come to the DRC from neighboring countries, including Rwanda, the Central African Republic, and Burundi [10]**.** ​Despite vast natural resources, widespread poverty persists in the DRC as evidenced by the low human development index (HDI) [11]. The DRC also failed to meet any Millennium Development Goals**,** though it has seen significant health improvements since 2000 as indicated by the increase in contraceptive prevalence and the national increase in immunization coverage (despite sub-national variability [12]) which has resulted in an overall decrease in the infant mortality rate [13]. In recent years, iterative Ebola epidemics have also plagued the health system, with the 10th ongoing epidemic beginning in August 2018 declared an international health emergency in 2019 [14].

The DRC was declared free of wild poliovirus (WPV) in November 2015 after four years without a case of WPV, and having met certain programmatic thresholds for immunization coverage (at least 90% third dose oral polio vaccine (OPV3) coverage), AFP surveillance (at least 3 cases per 100,000 persons detected), and given the existence of containment plans. The DRC, however, remains affected by four strains of cVDPV (type 2) in four provinces (Haut Katanga, Mongala, Haut Lomami/Tanganika/Haut Katanga/Ituri, and Kasai), with 82 confirmed cases in 2019 and 20 in 2018 [15]. Low routine immunization coverage, especially coverage of Inactivated Polio Vaccine (IPV) through routine service delivery mechanisms since the cessation of type 2 oral poliovirus, continues to challenge DRC’s eradication efforts and continue reliance on polio campaigns [16, 17], while the timeline, process, and indicators by which cVDPV eradication will be measured by the GPEI remains unclear.

### Ethiopia

With a projected population of 98.6 million in 2019 [18], Ethiopia is the second most populous country in Africa next to Nigeria. It is one of the least urbanized, however, with less than 20% of the population living in urban areas [18]. Ethiopia shares a long boundary with Somalia, Kenya, Sudan and South Sudan and as a result, experiences significant cross-border population movement. (While the country also shares a long boundary with Eritrea, the border was closed because of the Ethiopia-Eritrea border war until much recently, and there was little to no cross-border population movement). Ethiopia has seen significant health improvements in the last several decades, including a drastic reduction in under-five mortality (59.6% decrease between 2000 and 2016) [19]. These improvements were facilitated by an expanded primary health care system, including the introduction of a health extension program (HEP) in 2003, and an increase in immunization coverage [19]. Despite overall improvements, equity is a challenge in Ethiopia, where significant regional health disparities persist, and the country remains relatively dependent on external funding to finance its health services. Lowland, peripheral areas with pastoralist and semi-pastoralist populations, including the Afar, Gambella, Oromia and Somali regions, experience particularly poor EPI service coverage. In these regions, coverage of the third dose of polio vaccine among children 12–23 months was 25, 57.2, 53.8 and 26.8%, respectively, as compared to 93.1% in the Addis Ababa capital region [20]. The population in the Somali Region of Ethiopia is also highly mobile moving between districts, called *Wordeas*, regularly, making service delivery particularly challenging. Communities in this region have been chronically underserved by formal health services.

The last imported case of WPV in Ethiopia was in the Somali region in 2014. Ethiopia was removed from the list of polio-endemic countries by the WHO in 2015 and in June 2017, was recognized by the African Regional Certification Commission (ARCC) for maintaining polio-free status for nearly four consecutive years, though it maintained a “key at-risk” classification from the GPEI in part because Ethiopia is located in the wild poliovirus importation belt, a band of countries in Africa which are recurrently re-infected, where any pockets of low coverage as well as suboptimal surveillance system performance can make Ethiopia susceptible to outbreaks should importation occur. More recently in May 2019, Ethiopia was reclassified as an outbreak country given confirmation of an isolated cVDPV2 virus associated with an outbreak in the Horn of Africa, and the presence of cVDPV3, both in the Somali region [21].^1^

## Methods

The STRIPE project utilized a sequential, explanatory mixed-methods design to map tacit knowledge, i.e. the ideas, approaches and experiences from implementation which are not documented but are relevant to understanding both intended and unintended results of the program [22, 23], derived from the polio eradication experience from 1988 to 2019 [24, 25]. This included two methods of data collection: a quantitative survey followed by key informant interviews (KIIs). The survey was utilized to identify key themes related to implementation barriers which occurred in both settings. Analysis of the KIIs was organized according to these themes to further describe implementation barriers, strategies for addressing these challenges, and their intended and unintended outcomes across different socioecological levels according to the implementers’ perspectives [26]. The survey respondents were selected from a universe of polio workers identified in each country. The universe was defined as individuals who have been working continuously in the polio program for 12 months or more – and were identified from GPEI organizations, Ministry of Health personnel, NGOs and key agencies providing health services at different levels in both countries. The methodology for the STRIPE project, including the sampling methodology and respondent selection process, is described in detail in two other papers that are part of this series [7, 27]. Table 1 (below) describes respondent characteristics for both the survey and KIIs.

**Table 1:** Characteristics of Respondents^a^

	Democratic Republic of Congo		Ethiopia	
	Survey (***n*** = 499)	KIIs (***n*** = 23)	Survey (***n*** = 101)	KIIs (***n*** = 30)
**Level worked**^a^				
National	52 (10.4%)	13 (56.5%)	9 (8.9%)	7 (20.0%)
State/District	398 (79.7%)	7 (30.4%)	81 (80.2%)	17 (26.7%)
Sub-district/Frontline	256 (51.3%)	3 (13.1%)	44 (43.6%)	6 (53.3%)
**Organization**				
Government	109 (21.8%)	11 (47.8%)	113 (111.9%)	18 (60.0%)
GPEI Partners^b^	167 (33.5%)	7 (30.4%)	16 (15.8%)	4 (13.3%)
Implementing organizations/NGOs^c^	93 (18.6%)	2 (8.7%)	22 (21.8%)	5 (16.7%)
Research/academic organizations	6 (1.2%)	–	2 (1.9%)	3 (10.0%)
Other	67 (13.4%)	3 (13.1%)	24 (23.8%)	–

^a^Totals exceed 100% because many respondents worked at multiple levels, with multiple organizations over time. ^b^GPEI partners include the World Health Organization, the United Nations Children’s Fund, Rotary International, the U.S. Centers for Disease Control and Prevention), and the Bill and Melinda Gates Foundation. ^c^Implementing organizations/NGOs includes non-government, non-GPEI organizations involved at all levels

In the survey, we measured implementation barriers according to constructs within an adapted version of the Consolidated Framework for Implementation Research (CFIR), which assesses internal and external contextual factors that influence program implementation [28]. In Ethiopia, survey respondents were identified by snowball sampling from selected study areas. In the DRC, an online survey was distributed to national-level actors for which an existing sampling frame of immunization system actors was available; district level survey respondents were identified by randomly selecting health zones. In both Ethiopia and the DRC, survey questionnaires were administered as facilitated/in-person surveys where the study teams were unable to collect online responses [29].

The survey data was extracted and cleaned in R (version 3.3.2) [30] and exploratory data analyses summarizing the key variables including background characteristics of respondents, the polio program goals in different contexts, contextual factors that were barriers to the polio eradication activities, and the strategies that were deployed to address these barriers were conducted in STATA I/C (version 14) [31]. In analysis we also organized challenges by the level at which they occurred, e.g. cross-cutting process barriers, environmental, health system/organizational, and community levels.

The key informant interviews (KIIs) were administered to a nested sample of survey respondents selected to ensure representativeness across subject areas and barriers identified in the survey. Respondents held roles in government, the GPEI, and other implementing organizations. For logistical reasons, the DRC team conducted interviews in the capital city of Kinshasa; in Ethiopia, 50% of interviews were conducted in high-risk areas of polio transmission (Somali, Gambella and Benishangul Gumuz Regions) and the remainder in Addis Ababa City Administration and Oromia Regional State. The KIIs were guided by an interviewer guide which included prompts related to polio program organization and change over time, implementation context, challenges, and strategies, and lessons learned.

In Ethiopia, the KIIs were conducted by six data collectors who were Masters of Public Health graduates and who received three days of training on qualitative data approaches. In Ethiopia, the interviews were conducted in Amharic, voice recorded, and then then transcribed and translated to English for coding in Dedoose (version 8.2.31). In the DRC, interviews were conducted by individuals with medical and Master of Public Health degrees after a 5-day training. Interviews were conducted in French and the same process for recording and transcription, translation, and analysis was followed.

The background characteristics of KII respondents were summarized and both deductive and inductive data analyses implemented. First, the data was summarized according to constructs from the Consolidated Framework on Implementation Research (CFIR) [28], the socioecological model (SEM) [26], the PESTLE mnemonic,^3^ and the health systems building blocks [32]. The data was also inductively analyzed for emerging themes around barriers to polio program goals, strategies for addressing those barriers, and the intended and unintended outcomes of those strategies. The data summaries from both deductive and inductive analyses were used to describe a case for the polio program successes and failures in each country noting the barriers and strategies, and also incorporating data from the quantitative analyses and literature review. These variables served as the basis for a cross-case comparison between the DRC and Ethiopia.

The study protocols for each country (DRC and Ethiopia) were reviewed and approved by the Kinshasa School of Public Health Institutional Review Board and the Institutional Review Board of the College of Health Sciences of Addis Ababa University. Both survey and KII tools for data collection were pilot tested to ensure feasibility, utility and clarity of the data collection tools. The finalized tools were translated into Amharic in Ethiopia and French in the DRC. Surveys were conducted during November–December 2018 in Ethiopia, followed by the KIIs in December–January 2019. Surveys were conducted August 2018–March 2019 in the DRC, with interviews conducted from January–March 2019.

## Results

Table 1 above includes data on survey and KII respondents; it is worth noting that many respondents had held multiple roles at different levels of the health system. In both the DRC and Ethiopia, the highest percentage of survey respondents worked at the state/district level, at 79.4 and 80.2%, respectively. Given that the polio program was embedded into existing EPI structures in both countries at the operational/district level, the majority of survey respondents were employed by the government. The average number of years survey respondents worked in polio eradication was 8.24 (SD = 606) and 10.0 (SD = 5.64) years for the DRC and Ethiopia, respectively.

Respondents worked on a range of programmatic goals, with the highest proportion involved in vaccination (63.13 and 67.33% in the DRC and Ethiopia, respectively), followed by surveillance (44.69 and 42.57%), community engagement (31.26 and 27.72%), immunization systems strengthening (25.85 and 22.77%), and monitoring and evaluation (18.84 and 27.72%). A more limited sample worked in the areas of resource mobilization and partnership and strategy development.

### Implementation barriers

Survey results from the DRC and Ethiopia are presented in Table 2 below according to the CFIR implementation barrier types [28]. External settings, that is environmental factors that are outside of the program activities (e.g. political changes, social upheavals, geographical inaccessibility, economic and infrastructural challenges), but which bear influence on program implementation were identified as major implementation barriers by almost all respondents for different polio program goals (e.g. strengthening delivery systems, surveillance, monitoring and evaluation). In both the DRC and Ethiopia, survey respondents viewed these factors as the most significant barriers to polio eradication (41.15 and 53.20% of barriers identified, respectively). This sentiment was echoed by KII respondents who also highlighted the impact of external environmental barriers, along with health system barriers, on implementation processes. These cross-cutting barriers related to the process of conducting eradication activities (i.e. planning, executing, evaluating, and adapting) manifested across individual, community, and organizational levels to impede program implementation. We have focused our analysis here on the barrier types that were identified as the most significant by a majority of country respondents.

**Table 2:** Barriers to Implementation based on CFIR framework

		DRC	Ethiopia
Barrier Type	Definition	Number (%) of barriers identified (*n* = 1390) ^a^	Number (%) of barriers identified (*n* = 297) ^a^
Program characteristics	Activities conducted to enable polio eradication, including technologies adopted	84 (6.04%)	39 (13.13%)
Process of conducting the activities	How activities were implemented, including planning, execution strategies, evaluating and reflecting, adjusting and engaging	293 (21.08%)	39 (13.13%)
Characteristics of individuals	Characteristics of individuals within an organization involved in polio eradication activities	300 (21.58%)	36 (12.12%)
Organizational settings	Factors related to the organization(s) supporting the polio eradication program	141 (10.14%)	25 (8.42%)
External settings	Political, economic, social, technological, legal, and other environmental factors	572 (41.15%)	158 (53.20%)

Pearson’s Chi^2^ with 4 degrees of freedom = 44.66, *p* < .001

^a^The structure of the survey allowed a single respondent to reflect on barriers to multiple polio-related activities and also to identify multiple barriers for each goal. The DRC survey reached 550 respondents, who were involved in 1106 polio activities and identified a total of 1390 barriers (1.3 barriers per activity). The Ethiopia survey reached 109 respondents, who were involved in 212 polio activities and identified a total of 297 barriers (1.4 barriers per activity)

#### Barriers related to the process of conducting polio program activities

Cutting across levels of the socioecological model are challenges related to the process of conducting polio eradication activities [33]. These process barriers accounted for 21.08 and 13.13% of all barriers identified by DRC and Ethiopia surveys, respectively (see Table 2). Using the CFIR nomenclature, these process barriers can be further disentangled into barriers related to planning activities, executing activities, and monitoring and evaluating the activities. Of these process barriers, the majority – 42.08% in the DRC and 31.17% in Ethiopia – were related to execution (carrying out the activities according to plan, or program fidelity), followed by planning (the degree to which methods and activities are developed in advance) with 21.51 and 25.97%, respectively. One respondent explained this simply, noting that sometimes “the design is positive, but the implementation is defective” (national actor, DRC). Analysis of the KIIs suggest these processes were directly impacted by barriers at other levels, e.g. environmental, system, and community-level barriers, and are shaped by the characteristics of the individual implementer, e.g. leadership, vision, preparedness, motivations and attitude (which maps to the individual characteristics barrier type within the CFIR framework (Table 2)). For example, how human resource shortages contributed to individual barriers, and ultimately reduced fidelity of program delivery:

*“There are situations at which the activity was become monotonous for us. Due to [it becoming] tiresome there was a situation in which we take rest in between campaigning. This tiresome [feeling] happened due to high shortages of human power at a time. To speak truly there was time when one or two person vaccinates large kebele. Within this there was situation in which some children might be missed from vaccination.” -Frontline actor, Ethiopia*

This challenge reflects the sometimes-difficult conditions for implementers involved in polio eradication which could be de-motivating to staff, as well as lack of accountability mechanisms informing individual performance. Ensuring fidelity of program activities and improving planning mechanisms in the face of environmental and system challenges became a key strategic focus for the GPEI, and where these challenges were underestimated or insurmountable, it resulted in delayed program progress and increased risk.

#### Environmental barriers

Geographic inaccessibility emerged as a key challenge for polio eradication and health delivery equity in both the DRC and Ethiopia, compounded by broader economic and infrastructural challenges, shaped by the socio-political contexts. One DRC respondent explained, to enable implementation of vaccination and other programs the *“country must reach a certain stage of development; roads must be built; the waterways must exist”* and given “*the strong dependence on external financing, that means that we do not always know how to achieve the objectives we have set”(national actor, DRC).* Predictably, economic development influenced both existing infrastructure and the country’s ability to invest to overcome sizable implementation challenges, reflected in the survey responses which cited economic factors among the highest external barriers to implementation (28.14% in DRC, 20.43% in Ethiopia). The remote nature of some regions in Ethiopia (Somali, Gambella, Afar) also made it challenging to conduct vaccinations and surveillance, especially given the nomadic and semi-pastoralist nature of some communities. This and other issues of population movement in both countries – in/out migration, internally displaced persons in the DRC, refugees arriving in both Ethiopia and the DRC – posed significant challenges for implementers attempting to locate and reach mobile, often inaccessible communities, and reflected the complex socio-political forces at play.

Relatedly, issues of political insecurity directly disrupted program implementation. This was true to some extent in Ethiopia where intermittent protests, insecurity and inter-ethnic conflict occurred, but was particularly challenging in the DRC. Conflicts like the Kamwina Nsapu rebellion in the Kasai region paralyzed polio and other health-related activities for some time after the destruction of hospitals and health centers and given ongoing clashes between the government and Kamwina Nsapu’s followers, health workers’ access was often compromised. These clashes reflected strained relationships between the government and the population, raised issues of mistrust, and also challenged community acceptance of government-delivered health programs. One subnational DRC respondent described the impact of this for implementing organizations working in the region, saying:

*From the social point of view … I can say it is more in the corner where there is insecurity, where there are massive displacements, massive displacements of the populations … we do not know how to plan well. When we plan sometimes there are insecurities, there is incursion of armed groups, it also creates problems.* – Subnational actor, DRC

#### Health system barriers

Across the health systems building blocks, issues related to human resources, supply chain, finance, and governance emerged most strongly from the KIIs for both the DRC and Ethiopia and are underpinned by multiple CFIR constructs measured by the survey.

The DRC and Ethiopia both encountered challenges related to health worker shortages, limited health worker capacity, high turnover, and in some instances, lack of financial motivation and lack of supervision served as disincentives for health workers. One respondent summarized a sentiment shared by many implementers that human resource issues – tension between sects within the ministry, low pay, poor skill distribution – reflected systemic challenges, explaining, *“Today the health care organization in the country it ... it is fragile, it is weak, people are not paid and people are unstable”(national actor, DRC).* As another noted, *“personnel instability did not facilitate the smooth organization of polio immunization activities,”* and in fact challenged organizers’ ability to implement program activities consistently *(national actor, DRC).*

Tackling these issues proved difficult given insufficient health financing and, in the DRC, sometimes inappropriate diversions of financial resources. In Ethiopia, issues of poor financial flow led to disruptions in implementation and required adaptations in the field and over time, the ramp down of GPEI financing threatened sustainability of core activities. Working conditions were compounded by the hard-to-reach environments discussed above, which contributed to long hours for health workers and necessitated increased monitoring and supervision.

Regarding supply chain, respondents in Ethiopia noted recurrent vaccine stock outs in peripheral areas and in both settings, respondents discussed challenges in maintaining the cold chain given infrastructure and logistics management gaps. While these challenges were well known, lack of monitoring of the logistics system caused supply obstacles to persist over time, and per respondents in Ethiopia, required focused training of subnational implementers responsible for routine immunization as well as polio campaigns. Disruptions to cold chain infrastructure were also exacerbated by conflict as some refrigerators were destroyed in areas of the DRC.

These challenges reflected the organizational setting in each country, including its governance structures and mechanisms. In the DRC, among organizational-level barriers implementation readiness was the most often cited issue among survey respondents. This included a lack of leadership engagement, a lack of availability of resources, and limited access to knowledge and information. KII respondents also discussed limited engagement from the DRC government in support of the GPEI at various stages of the eradication effort given competing priorities, as well as challenges in financial and managerial accountability, particularly after decentralization reforms. One respondent described the challenges associated with creating “harmony” between each level of the health system, explaining,

*There is a tendency to say that polio eradication activities start from the top to the bottom, which is not true … because in order for the summit to put all the needs together and try to mobilize the resources, it is necessary that the information comes from the base. The same is true for all activities … - National actor, DRC*

In both settings, implementation readiness was partially dependent on the nature and quality of communications (“networks”) and capacity for change and receptivity to the intervention (“climate”), so far as these facilitated, or failed to facilitate, policy transfer among some stakeholders. One respondent from Ethiopia discussed the absence of these mechanisms, explaining:

*There is nobody to take the responsibility on calling for meetings, discuss [ing] things to work on together, and solve problems too. For example, if one partner has a problem, there might be a condition where the other partner covers his position if they are on the same area … We would also share knowledge on how they implement, and how I implement. If we talked about those things, we would gain knowledge. Then, it will gradually accumulate and become a national asset … But, there is no opportunity to create this kind of environment, no condition. – Subnational actor, Ethiopia*

Taken together, barriers related to system inputs (e.g. health worker shortage) and system functions (e.g. communications among health personnel) negatively impacted program fidelity and slowed progress toward GPEI goals.

#### Community barriers

At the community level, social challenges were the most commonly cited of all barriers identified by survey respondents, and key informants discussed program acceptability issues at length. Acceptability of polio vaccination was generally high in both settings, and social resistance was isolated to small geographical and cultural groups. Still, from the implementer’s perspective, this was an outsized barrier to implementation because it was exceedingly difficult to address, and threatened program success as near perfect coverage was required to achieve eradication. As one respondent from the DRC explained:

*The biggest challenge? Ah! The big challenge is the refusal … Because the child will not be vaccinated. When someone refuses to vaccinate their child … it’s someone you have to follow in such a way that they have to accept the vaccine. Other challenges are challenges, but solutions are easily found relative to the refusal because you have to make great efforts for them to accept. – Frontline actor, DRC*

Reasons for resistance across contexts were wide-ranging, reflecting how appropriate the community deemed the intervention (e.g. preference for injectable over oral vaccines in Ethiopia) as well as how the population perceived the implementers (e.g. mistrust of government in the DRC). In both settings, respondents attributed low demand to community fatigue given repeated campaigns, and among some community members, concern that health workers’ repeated visits were motivated by self-enrichment as each visit was an opportunity to generate benefits. As many respondents noted, global level stakeholders significantly underestimated the effort that would be required where community perceptions needed to be changed, and at some stages, missed the opportunity to implement best practices (see Strategies below) which could have prevented loss of community acceptance.

### Implementation strategies

KII respondents were asked to reflect on the implementation strategies utilized to address the barriers identified in the survey across levels. A summary of the strategies identified in each country are provided below (Table 3) according to the Expert Recommendations for Implementing Change (ERIC) framework on implementation strategies, which provides a consolidated compilation of implementation strategies with common nomenclature, definitions, and categories [34].^4^

**Table 3:** ERIC-Derived Implementation Strategies Utilized to Address Barriers to Polio Eradication

Implementation Strategy Type	DRC	Ethiopia
**Planning and resource mobilization**		
Develop a formal implementation blueprint	Develop microplans and promote bottom-up planning	Develop microplans and promote bottom-up planning Develop and utilize planning tools, e.g. integrated activity reports, training manuals, standard operating procedures, risk analyses
Acquire additional funding to facilitate implementation	Advocate government to set-up budget line for polio program Mobilize resources from local partners Utilize non-polio funding, e.g. Gavi grants, to cover cost of infrastructure improvements	Utilize non-program funds as stop gap until reimbursement possible Mobilize resources from local partners
Change service sites to increase access	Conduct mobile polio campaigns; set-up satellite sites under the supervision of rotating nurse	Conduct mobile polio campaigns in high population-movement zones Conduct frequent campaigns at border areas, in geographically inaccessible districts
Other	Not identified	Adjust dates, timing of campaigns based on available financial resources, vaccine supply
**Management and problem-solving**		
Assess organizational ability and readiness	Organize program review meetings to analyze program results and pitfalls, and come up with solutions	Not identified
Adapt physical structure and equipment to interventions	Not identified	Build and use solar refrigerators to ensure cold chain effectiveness
Build robust record systems to capture outcomes	Not identified	Leverage digital solutions to send reports from health facility to district and zone levels Utilize GPS technology to monitor community health worker activities at district and community level Utilize ODK systems to enable surveillance reporting in hard-to-reach areas
Centralize assistance for implementation issues	Not identified	Maintain frequent contact between regional health bureaus and federal ministry of health to manage problems as they occurred
Offer incentives or disincentives to providers and consumers	Use fiduciary agencies	Integrate health services, e.g. measles, tetanus vaccinations, newborn care, vitamin A supplementation, with polio campaigns Increase pay and compensation for health workers on campaigns, i.e. via stipends, materials, trainings
**Monitoring and evaluation**		
Develop mechanisms for feedback, monitoring and evaluation	Strengthen the national information system by establishing report analyses at each level, providing feedback for improvement	Conduct post-campaign evaluations to inform follow-up implementation activities Develop and conduct regular technical assessments at various levels of the health system
Conduct cyclical small tests of change	Not identified	Conduct regular review meetings to assess implementation status and performance, course correct
**Engagement and capacity-building**		
Build multidisciplinary partnerships and coalitions (to share knowledge, resources)	Not identified	Build partnerships to enable cross-border collaboration among health workers, volunteers, border security and immigration authorities, local leaders, including forming a cross-border health committee
Leverage existing collaborations and networks	Support international efforts to halt armed and inter-ethnic conflicts	Notify regional authorities of upcoming campaigns and request support, including obtaining support letters
Conduct workshops (to educate stakeholders, provide feedback or iterate program implementation processes)	Provide on-the-spot supervision to health workers conducting polio eradication activities to course correct, ensure fidelity	
Involve stakeholders, workers and consumers in the implementation effort	Engage peacekeeping troops in transport of vaccines to insecure zones	Engage schoolteachers in community mobilization, polio campaigns, community-based surveillance Utilize transport mechanisms from other sectors, traditional means of transport to facilitate campaign delivery
Recruit, designate and train leaders	Conduct continuous human resource training to build a pool of qualified candidates Train health workers in social mobilization	Capacity building of existing health professionals via in-service training Recruit health extensions workers, community volunteers to conduct vaccination, social mobilization, community-based surveillance
Promote supervision	Use polio resources to improve supervision of other activities	
**Communication and advocacy**		
Identify and prepare champions and early adopters	Advocate to actors at all levels of the health system, as well as opinion leaders, political leaders, notable persons/celebrities Involve members of parliament in polio program to garner support, including setting up parliamentary committee for immunization advocacy	Engage religious leaders as liaisons with community to increase community awareness and participation
Increase awareness among the population	Sensitize communities about benefit of immunization through social communication	Conduct intensive health education activities regarding importance of repeated polio doses, IPV

#### Strategies to address process-level barriers

Building robust record systems to capture outcomes, developing a formal implementation blueprint, developing mechanisms for feedback, monitoring and evaluation, conducting cyclical small tests of change, and promoting supervision were all identified as key strategies for addressing process barriers, and were also seen as a response to the system-level barriers experienced. Examples included conducting post-campaign evaluations, conducting regular technical and performance assessments, developing micro-plans, and promoting bottom-up planning. KII respondents in both settings emphasized the importance of tying these mechanisms with opportunities for stakeholders to meaningfully utilize data for decision-making. One DRC respondent explained that reflecting on and addressing implementation barriers became a core part of program management:

*We had the time to share this experience with others because before each phase of the campaign there is a training where everything is approached; planning, strategies, techniques, lessons learned, logistics and mobilization. The evaluation shows us the weaknesses that allow us to do the training themes*. – National actor, DRC

These opportunities were also critical for escalating implementation issues where required. A respondent in Ethiopia described this process: “*As partners we discuss about the issue and report to the head of health bureau regarding challenges [and they] have regular meetings so that at that time they discuss about the issue and provide future direction to solve a problem” (subnational actor, Ethiopia)*. Per the respondent, this management step was key for on-the-ground implementers working to adapt to emerging challenges, and from our analysis, was an operational change facilitated by the polio program over time.

#### Strategies to address environmental level barriers

Implementers’ ability to address environmental level challenges relied on their ability to adapt the program in response to external conditions, and to engage appropriate stakeholders in these efforts. One common strategy deployed was to change service sites to increase access, e.g. conducting frequent campaigns in border areas, and forming mobile vaccination teams to conduct outreach in geographically inaccessible areas and among mobile populations. Where necessary, campaigns were rescheduled around conflict activity. Another key set of strategies was in engagement and capacity-building, including building multidisciplinary partnerships and coalitions, and leveraging existing collaborations and networks. For example, peacekeeping troops were used to deliver vaccines in some insecure areas. Elsewhere, health workers relied on support from security personnel and religious and clan members to facilitate service delivery, including drafting official letters of support on behalf of health workers. These strategies enabled implementers to work around inalterable environmental barriers to achieve implementation goals. Some implementers felt forging multisectoral relationships could pay dividends for future programming as well, explaining: *“The coordination activities, coordinating with different sectors created a good communication ground … to implement other activities besides polio. So, it created the opportunity for political leaders to organize and collaboratively work with different sectors”* to tackle complex challenges *(Frontline actor, Ethiopia).*

#### Strategies to address health system barriers

At the health system level, tremendous investment was made by partners and government to build capacity in issue areas, including investments in health system inputs. Key strategies included adapting the physical structure and equipment to meet the intervention’s needs (i.e. improving cold chain and surveillance infrastructure), and recruiting, designating, and training leaders (i.e. improving human resource capacity). These strategies were necessary precursors to enabling implementation, without which the system was unable to meet polio performance metrics for immunization coverage and surveillance.

Finally, to address underfunding both countries worked to mobilize resources from local partners and to utilize non-program funds to cover costs, at least as a stop gap. In the DRC, advocacy at the central government level was also deployed to increase budget for immunization services including polio.

#### Strategies to address community-level barriers

Social mobilization activities were a core function of the polio program in both the DRC and Ethiopia and per respondents, contributed to improved demand over the course of the eradication initiative. Specific implementation strategies cited were identifying and preparing champions and early adopters, involving stakeholders in the implementation effort through “health committees,” and increasing awareness among the population via community education and sensitization. In the DRC, respondents noted the importance of advocacy at different levels of the health system, including local and religious leaders who could encourage immunization and facilitate delivery efforts. One respondent described this strategy of identifying and engaging “champions” in detail:

*“An example is the involvement of the leaders of those who were resistant, the sensitization of the people who were at the head of resistance. So that we can understand why they do not want to get vaccinated, answer questions that they had for them to understand that what they had, for example that the vaccines are there to make the girls sterile something like that, once they understood, we took them in the team of social mobilisers. Those who went to sensitize others so that when the leader understood (is convinced), he could convince others. We used them to facilitate our work with their followers.” – National actor, DRC*

Across countries, it was noted that social mobilization expertise (including developing worker’s knowledge base and interpersonal skills, ensuring frequent encounters and responding to feedback and challenges) needed to be built among staff, and messages appropriately tailored to the context. In Ethiopia, use of health extension workers (that is, government-salaried community health workers) engaged in ongoing intensive health education, vital registration, and case detection activities was particularly effective for improving relationships with the community and ultimately increasing demand.

### Intended and unintended consequences

Data from the knowledge mapping indicates that in many regards, the polio program had a positive effect on the DRC and Ethiopia’s health systems. Many respondents explained how capacity-building efforts in the areas of planning and management, monitoring and evaluation, and communication and advocacy have transferred to other health program management. Strengthening the process of bottom-up microplanning and data analysis and utility, for example, has led to more efficient and effective resource allocation (both financial and human) in both countries. Likewise, partnerships with stakeholders at multiple levels – cross-border national actors, community leaders – have been leveraged to benefit other social initiatives. Respondents also discussed examples of polio funding supporting essential investments in infrastructure and human resources and sustaining key health system functions. In the DRC, resources from polio eradication were used to purchase materials and equipment, to make improvements to the surveillance system, and to fund core activities such as enumeration, data analysis, active disease surveillance, and health worker training. Similarly in Ethiopia, polio funding contributed to supply chain improvements, laboratory function, and human resource training.

The benefits of these gains were optimized where the polio program was integrated with other services, i.e. polio immunization functioned through the routine immunization system, or other health targets were added to polio activities. For example, DRC respondents noted the benefit to health service provision (antenatal care, nutrition, deworming, bed net distribution, Vitamin A supplementation) of integrating GPEI-funded supervisory activities with other health initiatives. Across the study countries within the STRIPE consortium, surveillance was an area of high integration; in Ethiopia for example, the polio program supported the development of the Integrated Disease Surveillance and Response (IDSR) system and contributed to outbreak management functions.

Conversely, where the polio program operated in parallel to existing structures, polio activities tended to compete with routine service delivery. Respondents noted a drain on health care workers given higher incentives and pay for polio workers in the DRC compared to government-funded health workers, and demands of polio activities (campaigns, trainings) drew health workers away from conducting regular tasks in both settings. In some instances, this led to the disruptions of services at health posts; up to a week in some areas of Ethiopia. The issue of differential pay extended to outreach workers; in the DRC, positions typically held by volunteers were paid by the GPEI, leading to a shift in expectations that similar roles in other programs be compensated, though the budget to do so was not consistently available. Per respondents, social mobilization activities focused on polio vaccination and did not explicitly work to generate demand for routine vaccinations.

This prioritization of polio activities and the allocation of financial, human and time resources that followed came at a cost to routine immunization, which was allowed to lag at times as polio efforts ramped up. It also created some fissures with communities, which distrusted frequent campaigns. In the DRC, some communities felt service providers may have been motivated by self-enrichment rather than community benefit and were increasingly frustrated with the emphasis on polio eradication which they deemed a lower priority than issues of economic deprivation and malnutrition, which contributed to mistust. In Ethiopia, while polio eradication activities contributed to community demand for immunization services, over time community members were fatigued by repeated campaigns and grew to expect other services be delivered in-home, creating unmet expectations between beneficiaries and the government. Importantly, though chronic issues which contributed to weak health systems and limited service access existed prior to the polio initiative, the disparity felt by communities between the well-resourced polio program and other health service provision deepened issues of mistrust among some populations, especially in cases where community members were skeptical of the skill level of health workers, or held preexisting concerns regarding government intentions.

## Discussion

The findings from the DRC and Ethiopia illustrate the profound impact of external factors on program implementation. In both settings, geographic inaccessibility, conflict, and mobile populations emerged as central challenges to ensuring delivery of the GPEI’s four-pronged strategy, though the root causes and manifestations varied. While these challenges could not be addressed directly, implementers were successful when they were able to be flexible and responsive to emerging issues, including adapting service delivery to meet beneficiaries where they were, and were able to develop sound management strategies for planning, monitoring, and evaluation. Across socioecological levels, these strategies were instrumental in ensuring activities throughout the implementation cycle were carried out effectively. Likewise, efforts to improve vaccine demand among hesitant communities were most effective where implementers were able to engage locally relevant stakeholders who understood local norms, could anticipate issues, and facilitate community acceptance through ongoing and responsive communication. Deployment of social mobilization tactics played a key role in addressing this barrier in both settings. Executing these strategies required extensive, ongoing investment, as did ensuring the health system could adequately support program activities. For the DRC and Ethiopia, addressing gaps in human resources for health and cold chain were particularly critical, as were improving governance structures and financial mechanisms.

System investments indubitably improved program delivery, though the net impact on the health system in each setting is less clear, and in some regards, remains to be seen. Previous studies throughout the WHO AFRO region have documented how polio eradication has contributed to specific health system issue areas including narrowing the health workforce gap [35], strengthening laboratory systems [36], and improving disease outbreak preparedness and response [37]. Our own study confirms these findings and suggests both hardware (e.g. equipment) and software (e.g. skill-building) have benefited other health program operations and may have led to some within-system capacity transfer. This is particularly true where the polio initiative was integrated into the existing immunization system and, as cited elsewhere, with other health initiatives like Ethiopia’s Health Extension Program [38]. Where this occurred, capacity-building of actors involved in polio eradication also bolstered the skill level of EPI personnel who have increasingly taken on health leadership roles, and functional improvements benefited immunization systems. The longevity of these gains, however, is at risk given a heavy reliance on external assistance and diminishing financial resources from the GPEI as primary programmatic objectives are reached, a concern which emerged strongly among DRC respondents who at times described the polio initiative as “partners’ work.” In addition, even with other investing streams (e.g. Gavi, the Vaccine Alliance) supporting routine immunization, the sometimes singular drive to meet eradication aims led to the de facto de-prioritization of routine immunization in both settings and, in some instances, the development of parallel structures (e.g. differential pay for health cadres, siloed information systems) which have proven difficult to integrate and which may have distortive effects on the health system in the years to come.

These findings point to a few key lessons for future health initiatives. First, health programs should approach strategy and programmatic development carefully, utilizing multidisciplinary teams within global institutions to conduct systematic assessments to map contextual challenges, evaluate the political economy of the implementing contexts, and determine program readiness. As indicated in the DRC case, interethnic conflicts, often entailing massive displacements of populations, can dramatically affect implementation; in Ethiopia issues related to in/out migration and the presence of pastoralist communities likewise challenged implementers’ ability to ensure coverage of hard-to-reach populations. While these phenomena are unpredictable by nature, issues related to political insecurity, fragility, and population movement must be part of scenario-planning throughout the implementation cycle. Similarly, socio-anthropological studies and health systems assessments are important precursors to implementation which can help detect potential implementation barriers, e.g. issues related to community mistrust, local and national politics, and governance and accountability. Incorporating these factors into global planning processes will be critical for enabling implementation, helping to identify stakeholders capable of facilitating implementation and opportunities for maximizing positive externalities within communities.

Second, future initiatives should consider ways to leverage program resources to improve health systems rather than draw internal resources away from other health priorities. In addition, programs, particularly vertical disease control programs, should be wary of creating alternate structures which temporarily maintain health system functions, without enabling the requirements for sustaining those functions once program goals are met. This is a particularly important consideration when working in contexts with relatively weak health systems. Failure to strengthen the existing system can jeopardize both the immediate success of the initiative and its long-term impact, especially where there is no clear strategy for transition. Today, remaining coverage gaps in routine immunization have left both the DRC and Ethiopia susceptible to cVDPV and risk reemergence of the wild poliovirus without continued investment in improving routine immunization and maintaining disease surveillance systems, and given insufficient institutionalization of polio-related assets. Focused efforts in these areas have the potential to optimize positive externalities of the polio eradication initiative on the health system while ensuring the GPEI achieves its final aim of global eradication. This will not be achieved, however, unless national actors have meaningful opportunities to push back when they see fragmentation occurring, and unless there is a clearly defined exit strategy from the outset of implementation.

Finally, the polio eradication experience in these two countries provides a promising roadmap for future health programs seeking to reach consistently inaccessible communities. A core set of strategies to improve enumeration, microplanning, data utilization, communications, and outreach were effective in both settings when deployed with fidelity, and in response to contextually defined barriers to implementation. These precision tools have the potential to reduce the know-do gap between those interventions which are efficacious, but which do not reach those in need, but only if they are deployed with purpose. Future global health programs should consider how these strategies can be incorporated into an equity-driven agenda to reduce health disparities, including reaching communities in Ethiopia and DRC with consistently low immunization coverage for non-polio vaccines.

### Strengths and limitations

An important strength of this study is the breadth and depth of data sources, encompassing respondents across levels and organizations. The systematic nature of the survey sampling methodology [24] allowed for more robust conclusions and importantly, both the quantitative and qualitative methods have leveraged tacit knowledge to generate findings which are salient for public health implementers [6]. The sequential mixed methods design [20, 21] further strengthened the study as we were able to utilize the large sample size of the survey to identify issues of significance, and then explore those findings in-depth through the KIIs to unpack the consequences of implementation barriers and stakeholders’ strategies to address them. Still, our study is not without its limitations. While we endeavored to speak with implementers with experience across time, our findings are subject to recency bias as some implementers with experience early in the initiative had left their posts, retired, or were otherwise unavailable at the time of data collection. Finally, our findings should be interpreted in concert with other epidemiologic and implementation data sources, e.g. grey literature. While our study teams made an effort to collect documentary data to support the primary data collection (results from which are addressed elsewhere in this series [39]), they faced some challenges given the mixed quality of documentation and reporting practices, inaccessibility to some grey literature, and limited scope for literature review as it pertained to this manuscript.

## Conclusion

The polio program has been successfully implemented in the DRC and Ethiopia to achieve WPV-free status despite environmental, system, and community-level barriers, barriers which continue to threaten the continued success of the program in both countries. Strategies to strengthen planning, promote accountability and learning, adapt programmatic activities, and engage with local communities were crucial in mitigating these barriers, and will continue to be relevant to maintaining success. Areas of low immunization coverage and gaps in surveillance persist in both countries and must be addressed in order to prevent importation of wild poliovirus and minimize circulating vaccine-derived poliovirus. With the benefit of hindsight, some of the barriers to the polio program may have been anticipated with extensive pre-implementation and readiness assessment with local stakeholders, and participatory planning. The prioritization of polio campaigns may have contributed to missed opportunities for effectively integrating routine immunization system for all childhood antigens, and routine immunization may have suffered at the expense of polio programming, though more research may be needed to explore this theme further. Future health programs should consider strategies which reduce parallel structures and limit external dependency and should consider equity-driven agendas which utilize the implementation tools, strategies, and principles from polio eradication to ensure delivery of basic health services among hard-to-reach populations.

## Data Availability

The datasets supporting the conclusions of this article are available via the Open Science Framework repository, https://osf.io/9mavk/?view_only=dcaefa4871f94a9cb37ed72c1d29dc4a.

## Abbreviations

AFP: Acute flaccid paralysis
ARCC: African Regional Certification Commission
BMGF: The Bill and Melinda Gates Foundation
CDC: United States Centers for Disease Control and Prevention
CFIR: Consolidated Framework for Implementation Research
cVDPV: Circulating vaccine-derived poliovirus
DRC: Democratic Republic of Congo
EPI: Expanded Program on Immunization
EPHI: Ethiopian Public Health Institute
ERIC: Expert Recommendation for Implementing Change
fMOH: Federal Ministry of Health
GPEI: Global Polio Eradication Initiative
HDI: Human Development Index
HEP: Health extension program
HiT: Health systems in transition
IDSR: Integrated disease surveillance & response
INRB: National Institute of Biomedical Research
IPV: Inactivated poliovirus vaccine
JHSPH: Johns Hopkins Bloomberg School of Public Health
KII: Key informant interviews
NGOs: Non-governmental organizations
NHA: National Health Accounts
OOP: Out of pocket [payment]
OPV3: Third dose oral polio vaccine
PESTLE: [mnemonic] political, economic, social, technological, legal, environmental
PHEM: Centre of Public Health Emergency Management
SEM: Socioecological model
STRIPE: Synthesis and Translation of Research and Innovations from Polio Eradication project
UNICEF: United Nations International Children’s Emergency Fund
WHO: World Health Organization
WPV: Wild poliovirus

## Footnotes

1 At the initiation of research, Ethiopia was classified as a “key at-risk” country, defined as vulnerable to polio reemergence given low levels of immunity and poor surveillance systems. However, in May 2019 after data collection was completed, a case of cVDPV2 was detected in the Somali region, linked to an outbreak in the Horn of Africa in 2018. Ethiopia was subsequently re-classified by the GPEI as an “outbreak” country while cVDPV remains. More recently in August 2020, the African Regional Certification Commission declared the WHO African Region wild poliovirus free after four years without a case. At the time of publication, both the DRC and Ethiopia, along with 14 other countries throughout the region, were affected by cVDPV.

2 Given the recent outbreak, the Ethiopian government and GPEI partners have re-shifted focus toward emergency response, including full field investigations and development of outbreak response plans.

3 PESTLE stands for political, economic, social, technological, legal, and environmental.

4 Notably, only those strategies which were identified as relevant to the polio program in the DRC and Ethiopia were included in Table 3, and thus not all strategies from the ERIC framework are enumerated.
